# Physicians’ brain drain - a gravity model of migration flows

**DOI:** 10.1186/s12992-019-0536-0

**Published:** 2020-01-14

**Authors:** Alina Botezat, Raul Ramos

**Affiliations:** 1 Romanian Academy, “Gh. Zane” Institute for Economic and Social Research, 2 Teodor Codrescu Street, 700481 Iasi, Romania; 2 AQR-IREA, University of Barcelona and IZA, Av. Diagonal 690, 08034 Barcelona, Spain

**Keywords:** Brain drain, Medical doctors, Gravity model, Pull factors

## Abstract

**Background:**

The past two decades have been marked by impressive growth in the migration of medical doctors. The medical profession is among the most mobile of highly skilled professions, particularly in Europe, and is also the sector that experiences the most serious labour shortages. However, surprisingly little is known about how medical doctors choose their destinations. In addition, the literature is scarce on the factors determining the sharp rise in the migration of doctors from Africa, Asia and Eastern and Southeastern Europe, and how the last economic crisis has shaped the migration flows of health professionals.

**Methods:**

We use the new module on health worker migration provided by the Organisation for Economic Co-operation and Development (OECD) for 2000–2016 in order to examine the channels through which OECD countries attract foreign physicians from abroad. We estimate a gravity model using the Pseudo-Poisson Maximum Likelihood estimator.

**Results:**

Our results reveal that a lower unemployment rate, good remuneration of physicians, an aging population, and a high level of medical technology at the destination are among the main drivers of physicians’ brain drain. Furthermore, an analysis of the mobility of medical doctors from a number of regions worldwide shows that individuals react differently on a country-wise basis to various determinants present in the destination countries. Physicians from African countries are particularly attracted to destination countries offering higher wages, and to those where the density of medical doctors is relatively low. Concurrently, a higher demand for healthcare services and better medical technology in the receiving country drives the inflow of medical doctors from Central and Eastern Europe, while Asian doctors seem to preferentially migrate to countries with better school systems.

**Conclusions:**

This study contributes to a deeper understanding of the channels through which OECD countries attract foreign medical doctors from abroad. We find that, apart from dyadic factors, a lower unemployment rate, good remuneration of physicians, an aging population, and good medical infrastructure in the host country are among the main drivers of physicians’ brain drain. Furthermore, we find that utility from migration to specific countries may be explained by the heterogeneity of origin countries.

## Introduction

The migration of tertiary educated people from poor to rich countries has become an increasingly important feature of international migration. Over the past few decades, the stock of highly-skilled immigrants in member countries of the Organisation for Economic Co-operation and Development (OECD) grew at a much faster rate than low-skilled workers [1]. The major determinants underlying this trend are: the worldwide expansion of education, declines in migration costs, and the increased role of human capital in economic development; all of which has led policymakers to increase attempts to attract specialised workforces from abroad [2].

One critical dimension of skilled migration, is the persistently higher mobility rates of health professionals. The medical profession is among the most mobile of highly skilled professions, particularly in Europe. It is also the sector that experiences the most serious labour shortages [3, 4].

However, surprisingly little is known about how medical doctors choose their destinations. The main reason for this knowledge gap is a lack of appropriate data. To the best of our knowledge, there are only three datasets on physician migration: First, the OECD Health Statistics - Health Workforce Migration dataset, which contains yearly observations on foreign-trained health professionals for the OECD countries, but the temporal coverage is unbalanced [e.g., for France: 2011–2014, for Germany: 2000–2014]. Second, the dataset of Clemens et al. [5], with data on the emigration of African physicians from nine destinations countries in 2000 [3]; the dataset of Bhargava et al. [6], which extends the database of Clemens and Pettersson [7] and includes observations from 18 additional countries from 1991 to 2004.

In our paper, we take advantage of the new module on health worker migration provided by the OECD for 2000–2016, in order to examine the determinants of migration flows of foreign-trained physicians. We employ a gravity-type estimation model using the Pseudo-Poisson Maximum Likelihood estimator, developed by Santos Silva and Tenreyro [8]. Our focus is on the pull factors, since the impact of push factors on emigration rates is comparatively small, as shown by Mayda [9]. In particular, Mayda finds a clear asymmetry between the push and pull factors that explain migration flows between 14 OECD countries and the rest of the world. While the statistical significance of pull factors is very robust to different specifications, the effect for push factors is much smaller and statistically insignificant in several models. He provides a tentative explanation: this result could be related to the moderating effect of destination countries’ migration policies, which can become more restrictive when migration pressures increase due to changes in pull factors.

Our paper’s contribution to the literature is threefold. First, while many studies document the effects of physician emigration on various health and socio-economic outcomes in the sending countries [6, 10–12], a much smaller subset focus on the determinants of the direction of migration flows for medical doctors. In one of very few studies which analyse the migration of medical physicians from 144 origin countries to 18 destination countries over the period 1995–2004, Yakovlev and Steinkopf [13] show that physicians are most attracted to countries characterised by greater economic freedom, a lower share of public health expenditures, and higher health spending per capita. Employing the same estimation strategy, our paper is most closely related to Moullan [14], who uses the data of Bhargava et al. [6] to study bilateral flows of foreign-trained medical doctors for the years 1991–2004. He finds that one of the main drivers is the healthcare market. Inflows of foreign-trained doctors are higher in OECD countries with a low density of doctors and with high social expenditures on healthcare. We add to this emerging line of research by investigating the factors determining the absorptive capacity of medical doctor inflows from abroad. Using recent data, 2000 to 2016, which covers the period when the migration of medical doctors increased significantly, we are able to provide new insights into the phenomenon of physicians’ brain drain.

In addition, very little is known about the factors determining the sharp rise in the migration of doctors from Africa, Asia, Eastern and Southeastern Europe, and how the last economic crisis shaped the migration flows of health professionals. Our paper fills this gap, and shows that utility from migration to a specific destination varies in magnitude both across categories of origin countries and over time.

Our research also adds to a new strand of literature that employs gravity models to investigate migration flows [15–22]. Among them, only a few use data on highly-skilled migrants. A notable example is Czaika and Parsons [19], where the authors use data for 10 OECD destinations and 185 sending countries over the period 2000–2012 to analyse which kind of migration policies favour the inflow of highly-skilled labour migrants.

## Material and methods

### Theoretical framework

In this section we briefly present the theoretical micro-foundations to the choice of destination country. By comparing costs with benefits, a rational individual will choose a destination that maximises utility. Formally, the utility function is specified based on the random utility framework introduced by McFadden [23]. This represents the starting point for the Random Utility Maximisation [RUM] models used in the migration literature to derive migration flow specifications. Following Bertoli and Fernández-Huertas Moraga [24], the basic RUM model takes the following form:

1 $$ {U}_{ijk}={V}_{jk}+{\epsilon}_{ijk}={w}_{jk}-{c}_{jk}+{\epsilon}_{ijk} $$

where *U*_*ijk*_ represents the utility of individual *i* after migration from country *j* to country *k. w*_*jk*_ represents the deterministic component of the utility, and *c*_*jk*_ the costs of moving from *j* to *k*. *ϵ*_*jk*_ is an individual stochastic component of utility. If it is assumed that *ϵ*_*ijk*_ has extreme value type-I distribution, we can apply the results in McFadden [23] and show that the probability of choosing destination k can be represented by:

2 $$ {p}_{ijk}=\frac{\exp \left({w}_{jk}-{c}_{jk}\right)}{\sum_{d\in D}\exp \left({w}_{jd}-{c}_{jd}\right)} $$

where *d* represents any possible destination out of the set of country choices *D*. Similarly, the probability of staying in the country of origin is given by:

3 $$ {p}_{ijj}=\frac{\exp \left({w}_{jj}\right)}{\sum_{d\in D}\exp \left({w}_{jd}-{c}_{jd}\right)} $$

Now we calculate the odds ratio of migrating to *k* versus staying in *j*:

4 $$ \frac{p_{ijk}}{p_{ijj}}=\frac{\exp \left({w}_{jk}-{c}_{jk}\right)}{\exp \left({w}_{jj}\right)}=\exp \left({w}_{jk}-{c}_{jk}-{w}_{jj}\right) $$

Thus, the odds ratio of migrating to *k* versus staying in *j* will depend on the attractiveness of the destination country (*w*_*jk*_), the costs of migration (*c*_*jk*_), and on the foregone utility of staying in country *j* (*w*_*jj*_).

But in reality, the decision to migrate is not based solely on push and pull factors present in the source and destination countries, but also on the relative opportunities and/or obstacles that other possible destinations simultaneously exhibit. In economics, Anderson and Van Wincoop [25] first formalised this idea in the gravity model of international trade as ‘multilateral resistance’. In the migration literature, Bertoli and Fernández-Huertas Moraga [24] introduced this term, referring to the” multilateral resistance to migration” as” *the influence exerted by the opportunities [barriers] to migrate to other destination*s”. Hence, as shown by Bertoli and Fernández -Huertas Moraga [24], the denominator in [2], which captures the attractiveness of other destinations, should depend on *k* as well.

Failing to control for these ‘third-country’ effects in the estimation of a gravity model of migration might lead to biased results. The influence of alternative destinations can confound identification of the effects of various determinants on the bilateral migration flows. In this sense, various empirical approaches have been proposed to tackle the problem (for an overview, see Head and Mayer [26] or Ramos [27]). For instance, Bertoli and Fernández-Huertas Moraga [24] and Bertoli et al. [28] suggest using the Common Correlated Effects (CCE) estimator of Pesaran [29] to account for potential biases due to multilateral resistance. This technique is particularly suitable when the data set is very large in terms of bilateral observations and time periods. But the most common approach in the literature is to use fixed-effects methods to control for multilateral resistance to migration. Bertoli et al. [28] include dyadic fixed effects and time fixed effects in the specifications to get unbiased estimates. Much less demanding in terms of data are the solutions proposed by Ortega and Peri [21] and Beine and Parsons [30], who use origin-year fixed effects or destination-year dummies.

For this reason, we follow the methodology applied by Ortega and Peri [21], and use origin-time fixed effects (see Eq. 5). Since our primary focus is on the ‘pull’ factors of migration determining choice of destination, by using origin-year dummy variables we are able to control for all” push” determinants present in sending countries.

### Estimation strategy

This section explains the econometric framework used to estimate bilateral flows of foreign-trained physicians through a gravity model approach. The estimable equation is as follows:

5 $$ {n}_{jk t}={\beta}_1\left(\ln {X}_{k,t-1}\right)+{\beta}_2\left(\mathit{\ln}{d}_{jk}\right)+{\beta}_3\left({C}_{jk}\right)+{\beta}_4\left({EU}_{jk t}\right)+{\beta}_5\left(\ln {m}_{jk,t-1}\right)+{\mu}_{jt}+{\delta}_k+{\epsilon}_{jk t} $$

The dependent variable *n*_*jkt*_ represents physicians’ migration flows from country *j* to country *k*. The subscripts *j*, *k*, and *t* refer to origin countries, destination countries, and time, respectively. The vector *X*_*k*, *t* − 1_ includes our main variables of interest related to a destination’s characteristics. For this, we include destination time-variant variables, lagged over one period. Here we refer to GDP per capita, unemployment rate, remuneration of physicians, and PISA reading score (as a proxy for the quality of the education system). Furthermore, we include variables describing a range of factors that affect both the supply of- and demand for health workers. By lagging these variables, we attempt to control for reverse causality [14]. Other controls include time-invariant bilateral factors, such as physical distance between origin and destination country (*d*_*jk*_), a common border, and cultural links (colonial heritage, common language, *C*_*jk*_). Two dummies that indicate EU and Schengen membership of pair countries are also included as controls. We add a variable for diaspora, defined as the stock of highly-skilled immigrants trained in country *j* and living in country *k* at the start of period *t*. A detailed description of the variables is presented in the next Section. Finally, we add time-varying origin dummies *μ*_*jt*_ to control for time-variant factors at origin, and destination fixed effects (*δ*_*k*_) to control for destination-invariant characteristics.

We estimate Eq. 5 by means of the Poisson-Pseudo-Maximum-Likelihood (PPML) approach proposed by Santos Silva and Tenreyro [8]. This method has several advantages. Firstly, a PPML estimator is fully consistent with the underlying random utility maximisation presented above. Secondly, the estimator is robust to different patterns of heteroskedasticity [8]. Thirdly, a PPML estimator is particularly suitable in regressions where the dependent variable has a significant proportion of zero values [30, 31]. In our case, 84.01% of our bilateral flows is equal to zero. Regarding the functional form, when using PPML, the dependent variable is in levels and all of the continuous independent variables are in logarithmic form (see Eq. 5). Aside from accounting for multilateral resistance to migration, the inclusion of origin-time fixed effects in Eq. 5 warrants that the Poisson-pseudo-maximum- likelihood estimates are always consistent with heterogeneity in the propensity to migrate [17, 32].

### Data and descriptive statistics

The data for the empirical analysis was gathered from various sources, and covers the time period 2000 to 2016. The origin and destination countries are listed in the Appendix. The selection of the variables is mainly based on previous studies of highly-skilled migration, physicians’ migration and the empirical specifications of the underlying random utility model (see Czaika and Parsons [19], Moullan [14], among others).

#### Dependent variable: immigration flows

Our key dependent variable is the dyadic inflow *n*_*jkt*_ and represents the inflow of medical doctors from country of origin *j* to country of destination *k* at time *t.* The data is provided by the OECD within the new module on health worker migration,^1^ which contains data on inflows and outflows of doctors and nurses across countries [33]. The OECD Health Migration Data has three main advantages. First, it covers all OECD countries as destination countries. Secondly, the data, jointly collected by OECD, Eurostat, and WHO-Europe is comparable across countries. The data is on foreign-trained doctors, representing the number of doctors who obtained their first medical qualification (degree) in another country and who are licensed to practice in the receiving country. This is particularly important, since focus on place of training is the most relevant for measuring ‘brain drain’ [33]. Finally, the data is reported annually, making it possible to monitor trends in health workforce migration on a yearly basis.

#### Explanatory variables

As destination-time-variant variables, we use the following. We include the ratio of GDP per capita for the country of origin relative to the GDP per capita for the country of destination, as a proxy for the gap in economic development and absorption capacity between both countries [34, 35]. A smaller ratio of GDP per capita might indicate a greater probability of obtaining higher income levels in the destination country [21, 30], which represents a key factor in the decision to migrate [36]. The data is taken from the World Bank.^2^

We also consider the unemployment rate in the destination country.^3^ Since employment exhibits a higher sensitivity to shocks than wages [16, 37], it represents a relevant ‘pull’ factor in the migration process; particularly for highly skilled migrants, given that a region which attracts an increasingly skilled inflow of migrants generally sees a higher average employment level [37].

We also control for quality of the education system in the destination country, given that physicians who consider emigration may also be concerned about the quality of education available to their children. For this, we use PISA reading score,^4^ since cognitive skills measured by PISA constitute a reliable proxy for school quality [38]. As PISA test scores are measured every 3 years, for those years we have no new scores available we assign the last available PISA test score (for years 2001 and 2002, the value from 2000; for years 2004 and 2005, the values from 2003, and so on).

In the literature, the existence of networks is expected to decrease migration costs, and favour migration flows [19, 39–41]. In order to control for network externalities, we follow Anjomani and Hariri [42] and include in our model the migration stocks of highly-skilled co-nationals from year 2000 (those with the same country of training, and who reside in the respective destination country). To do this, we use the Database on Immigrants in OECD Countries^5^ (DIOC), which includes information on place of birth and educational attainment.

Following the approach in gravity models of international migration, we control for time-invariant dyadic variables that also influence migration costs. For example, the physical distance between two countries is expected to be negatively correlated with migration inflows [43]. Likewise, linguistic proximity affects migration costs [44], particularly in the case of health professionals [45]. The measures of the distances between geographical regions, colonial relationships, official languages, and a common border are taken from the online database of the Centre d’Études Prospectives et d’Informations Internationales (CEPII).^6^

In addition, we entered two dummy variables for EU and Schengen membership, as a common membership to one of these agreements may facilitate the free movement of people [19, 21]. Of course, we take into account that some countries became EU and Schengen members in different years.

Given the purpose of the paper, we also look at the factors that describe the functioning of the health labour market in the destination countries. Following Moullan [14] and OECD [46], and given the available data, we consider the following factors affecting the supply and demand of medical doctors. The supply of health workers is mainly determined by the number of people practicing in the system. We control for the supply of medical doctors measured by the density of physicians per 1000 individuals.^7^ In addition, we also consider the number of medical graduates, defined as the number of students who have graduated from medical schools or similar institutions in a given year.^8^ The supply of medical doctors is also driven by the salary level. A higher salary may indicate an increase in hours for those working in the system, as well as an increase in the attractiveness of the medical profession. Given that higher-skilled workers are attracted by high wages [47], higher salary levels for medical doctors may also trigger an outward migration of health professionals from other countries. As an indicator for medical doctors’ pay, we use the remuneration for specialists, defined as the average gross annual income, including social security contributions and income taxes payable by the employee.^9^ Since data for this indicator is not available for all years in the OECD database, we also use data on average wages^10^ to compute the ratio between specialists’ wages and average years in a specific year and country, for which data is available. Using this ratio, and full data on average wages, we compute the remuneration of medical doctors for the missing years. In addition, we include a proxy for medical infrastructure and health care resources in a given country, as measured by the total number of computed tomography scanners per million individuals^11^ available each year in the health system. We expect a country with a rich medical infrastructure to be attractive to medical doctors from abroad. On the demand side of the health labour market, we consider the following variables: governmental health spending, health care coverage, age dependency ratio, and the number of hospital beds. Health spending per capita^12^ is an indicator of the capacity to pay health professionals from public resources, and is a good proxy for the degree of government involvement in the provision of healthcare. It is expected that countries for which health spending per capita is high will attract more medical doctors from abroad [13]. Health care coverage,^13^ measured as the share of the population covered by health insurance, is highly correlated with demand for health services. Similarly, the age dependency ratio,^14^ which is the ratio of older dependents (people older than 64) to the working-age population (aged 15–64), is an important demographic factor affecting the demand for health care services [13]. Finally, the number of hospital beds^15^ describes the physical health care infrastructure, and represents a good proxy for the inpatient sector [48].

Descriptive statistics on all variables are reported in Table 1.

**Table 1:** Summary statistics

	Observations	Mean	SD	Min.	Max.
Yearly Inflow	73,508	5.394	49.089	0	3640
Destination Controls					
Unemployment rate	70,688	7.464	3.237	2.558	26.117
PISA Scorereading	73,508	497.228	20.287	428	547
Dyadic Controls					
Ratio GDPo/GDPd	71,346	0.427	0.852	0.002	15.444
Stock of high-skilled migrants (2000)	71,924	7707.40	33,212.59	1	508,333
Distance	73,508	6812.444	4344.516	53.532	19,586.18
Colony	73,508	.041	.198	0	1
Common language	73,508	.120	.325	0	1
Contiguity	73,508	.019	.136	0	1
Both in EU	73,508	.061	.239	0	1
Both in Schengen	73,508	.056	.23	0	1
Supply-side Controls					
Density of physicians per 1000 population	68,996	3.13	.710	1.3	5.3
Medical graduates per 100,000 population	72,756	10.437	3.938	3.84	24.44
Remuneration of physicians (US$ PPP)	67,680	118,588.7	58,193.76	20,603.21	271,125.1
Medical technology	53,580	17.703	9.743	4.89	43.87
Demand-side Controls					
Public expenditures (US$ /capita)	73,508	3257.057	1644.86	425.6	9832.317
Health insurance coverage	70,124	98.528	3.909	69.8	100.2
Age dependency ratio	73,508	23.403	4.835	9.623	35.660
Hospital beds per 1000 inhabitants	70,688	4.849	1.866	2.05	9.12

The table reports summary statistics of the variables used in the gravity model

## Results

We estimate Eq. 5 in several specifications. The results are reported in Table 2. The baseline specification in column (1) only includes controls for destination and conventional dyadic factors. In model (2), we add control variables describing the supply of medical doctors. In column (3) we include the demand factors, while column (4) presents the results for the full model. Finally, in the last column we also include an indicator for medical infrastructure, for which we have fewer observations.

**Table 2:** Determinants of migration flows of medical doctors (2000–2016)

	Pseudo-Poisson Maximum Likelihood				
	(1)	(2)	(3)	(4)	(5)
Destination Controls					
Log Unemployment rate [t-1]	−0.091 (0.24)	− 0.669^c^ (0.18)	− 0.270 (0.22)	− 0.916^c^ (0.18)	−1.119^c^ (0.17)
Log PISA Scorereading [t-1]	−0.036 (2.34)	− 0.540 (1.97)	− 0.796 (2.20)	−0.901 (1.79)	4.894^b^ (2.17)
Dyadic Controls					
Log GDPo/GDPd [t-1]	−0.222 (0.34)	0.289 (0.34)	−0.037 (0.34)	0.932^c^ (0.34)	1.545^c^ (0.35)
Log Diaspora [2000]	0.004 (0.01)	0.004 (0.01)	0.014 (0.01)	0.014 (0.01)	0.034^c^ (0.01)
Log Distance	−0.691^c^ (0.07)	−0.697^c^ (0.07)	−0.847^c^ (0.06)	−0.864^c^ (0.07)	− 0.635^c^ (0.07)
Colonial-tie dummy	0.591^c^ (0.10)	0.600^c^ (0.09)	0.598^c^ (0.10)	0.613^c^ (0.09)	0.558^c^ (0.11)
Common language dummy	2.415^c^ (0.16)	2.375^c^ (0.18)	2.227^c^ (0.11)	2.168^c^ (0.12)	2.679^c^ (0.11)
Contiguity dummy	−0.205^b^ (0.09)	−0.265^c^ (0.09)	−0.266^c^ (0.09)	− 0.365^c^ (0.08)	− 0.135 (0.13)
Both in EU	0.097 (0.10)	0.205^a^ (0.12)	0.028 (0.10)	0.131 (0.11)	0.624^c^ (0.13)
Both in Schengen	0.712^c^ (0.11)	0.645^c^ (0.12)	0.750^c^ (0.09)	0.687^c^ (0.10)	0.276^b^ (0.14)
Supply factors					
Log Remuneration of physicians [t-1]		1.651^a^ (0.87)		1.998^b^ (0.91)	2.107^b^ (0.85)
Log Density Physicians per 1000 population [t-1]		−2.810^c^ (0.70)		−3.276^c^ (0.69)	−1.808^c^ (0.64)
Log Medical Graduates per 100,000 population [t-1]		−0.143 (0.35)		0.234 (0.34)	0.919^c^ (0.30)
Log Medical Technology [t-1]					1.033^c^ (0.40)
Demand factors					
Log public health expenditures [t-1]			−0.572 (0.63)	0.153 (0.64)	0.168 (0.51)
Log health insurance coverage [t-1]			−3.105^b^ (1.46)	−5.232^c^ (1.47)	−3.337^b^ (1.68)
Log Age dependency ratio, old [t-1]			4.853^c^ (1.10)	2.891^c^ (0.98)	4.684^c^ (1.01)
Log Hospital beds [t-1]			−0.410 (0.45)	0.803^a^ (0.43)	1.397^c^ (0.46)
Destination FE	YES	YES	YES	YES	YES
Origin-time FE	YES	YES	YES	YES	YES
Number of clusters (destination*time)	337	304	303	272	201
Observations	45,538	40,912	40,709	36,398	25,466
R-sqr	0.671	0.716	0.717	0.771	0.867

The table reports PPML estimates of the determinants of international migration in the destination country on the inflow of foreign-trained medical doctors. The dependent variable represents the number of foreign-trained physicians who have obtained a (partially or fully) registration to practice as medical doctor in the receiving country at time *t*

Estimation period: 2000–2016

Standard errors in parentheses are clustered by destination and time. ^a^, ^b^, ^c^ indicates significance at the 10, 5, and 1% level, respectively

The results reveal that, with the exception of GDP per capita ratio and PISA literacy score, the coefficients are similar in terms of magnitude and statistical significance across all specifications.

The coefficient estimate for the variable accounting for the unemployment rate in the destination countries is negative, and highly statistically significant in the full model. This indicates that the destinations with lower unemployment rates are those that attract an increased number of foreign medical doctors. Next, the results for our preferred specification (column 5) suggest that PISA test score, our proxy for education quality in the destination country, is positively correlated with higher inflows of medical doctors.

Further, results in columns (4) and (5) show positive and statistically significant effects of the GDP ratio on migration flows. In fact, we note that for specifications in which we control for remuneration of physicians (columns 2, 4 and 5), the estimates turn positive.

We also note that the stock of highly-skilled co-nationals in the destination country does not seem to matter for the next waves of physician inflows.

Next, we examine the influence of the standard dyadic control variables on migration flows. The results reveal that these variables all have the expected sign. The distance variable has a negative coefficient in all specifications, and all estimates are statistically significant and similar in magnitude. The estimated coefficient for *Log Distance* (last column) suggests that a 10% increase in bilateral distance is, on average, associated with a 6.35% reduction in migration flows of medical doctors. Further, a colonial relationship is positively correlated with migration flows of medical doctors. The language coefficients all have a positive sign, again as expected, and have the highest magnitude among all dyadic controls. The coefficient on *Common language dummy* from our full model (column 4), suggests that if two countries share the same language, they experience almost 800% higher bilateral physician flows than countries with different languages.

EU and Schengen membership leads to a positive increase in the immigration flows of doctors. The effect is highly significant, particularly for pairs that are both Schengen members. The coefficient from column 4 (full model) indicates that Schengen member countries experience 98.77% higher bilateral flows of medical doctors than non-Schengen countries.

A key rationale for attracting medical doctors from abroad is a response to shortages in medical staff [49]. For this reason, in specification (2) we include variables that describe the supply of medical doctors. The negative coefficients on density of physicians indicates that a low density determines an expansion of the health sector, also by attracting physicians from abroad. On the other hand, the density of medical graduates is positively associated with higher inflows of medical doctors (columns 4 and 5). The results also reveal a positive relationship between migration inflows of medical doctors and physicians’ remuneration in the destination country. The estimated coefficients (columns 4 and 5) suggest that a 10% increase in wages increases migration inflows of medical doctors by around 20%.

We further find that the various demand-side factors are differently correlated with physicians’ brain drain. Higher health spending per capita in destination countries does not have a conclusive effect on the number of migrant arrivals. A distinct pictures emerges when we look at health insurance coverage. It seems that a high share of the population covered by health insurance is not necessarily favourable for attracting medical doctors from abroad. The estimate is negative, and statistically significant across all specifications, even after controlling for supply factors. The coefficient of log age dependency ratio is positive, and highly statistically significant. Similarly, once we control for the supply factors, the number of hospital beds is significantly correlated with immigration flows of medical doctors, which indicates that inpatient sector infrastructure is correlated with the demand for medical doctors from abroad.

In the last column we also control for medical technology, as a proxy for health system infrastructure. We find a positive and highly statistically significant relationship between medical infrastructure and inflows of foreign-trained doctors. The results reveal that better health care resources in a medical system attracts physicians from abroad.

## Discussion

### Main results

This paper examines the channels through which OECD countries attract physicians from abroad. We show that a lower unemployment rate at the destination increases migration flows among medical doctors. This confirms previous empirical results, indicating that a higher employment rate is directly associated with an increasing trend in the volume highly-skilled migrants [24, 37]. Contrary to previous research on highly-skilled migration [19], our results also suggest that PISA test scores are positively correlated with greater inflows of medical doctors. Therefore, the number of foreign-trained physicians is much greater in countries that have better school systems. This finding is in line with previous studies on doctors’ motivations to leave their origin countries. A lack of good quality education in the home country [50] and a better quality of life [51, 52], including better education, are among the main reasons doctors choose to migrate.

We have also analysed the influence of the ratio of GDP per capita for the country of origin relative to the GDP per capita for the country of destination on migration inflows. While the GDP for the country of destination accounts for the incentive to migrate, the GDP in the home country reflects both the incentive to go abroad, and the ability to cover the costs of migration [41]. In this sense, we expected a positive effect when the ability to cover the migration costs dominates over the incentive effect, and a negative sign when the incentive effect is higher than the ability to finance the required investment. Results for our preferred specifications reveal positive and statistically significant effects of the GDP ratio on migration inflows. This suggests that if the ability to cover the costs of migration predominates over the incentive effect, with all else equal, the migration flows of medical doctors increase. Moullan [14] finds similar results, showing that a higher GDP per capita at the destination does not increase the inflows of medical professionals. This finding is also in line with previous results reported by Grogger and Hanson [53], Mayda [9] and Didisse et al. [54], who show how an increase in GDP per capita in the home country may increase emigration flows.

Furthermore, the diaspora in the destination country does not seem to matter for the next waves of physician inflows. This result is in line with those of Docquier and Rapoport [55], who argue that past physician migration does not influence the outward migration of medical doctors from the country of origin. Our results, however, contradict the evidence presented by Moullan [14], who shows that diaspora has a positive effect on the migration flows of medical doctors. This different result could be related to compositional effects, as his analysis refers to the period of 1991–2004 while ours focuses on 2000–2016. The difference could also be due to the fact that we consider a higher number of developed destination countries where network effects are usually less relevant [17]. Moreover, it has been shown that, in general, diaspora encourages the migration of low-skilled over highly skilled migrants [17, 40], and those from relatively poor origin countries [56].

The language coefficients are positive, and have the greatest magnitude among all dyadic controls; indicating that sharing a common language is a more important pull factor for medical doctors. This is very likely the case, since, in general, medical doctors willing to practice in another country must prove advanced language skills [3, 57]. But there also cases in which many foreign doctors face language problems in the host country [44], a relative lack of language skills being often perceived as a sign of a lack of medical knowledge [58].

Several studies examining the relationship between the characteristics of the health labour market and migration flows of medical doctors [14, 46] posit that supply and demand factors of healthcare professionals in the destination country are among the main drivers of physicians’ inflows from abroad. Our results indicate that a lower density of physicians in the destination countries determines an expansion of the health sector, by attracting physicians from abroad. This effect is slightly greater than the effect in Moullan [14], and is statistically significant even after controlling for the demand factors. On the other hand, we show that the density of medical graduates is positively associated with higher inflows of medical doctors. This result is in line with the findings of Moullan [14], who also observes a positive correlation. One explanation might be that the size of the tertiary education system is highly correlated with the country size, which, in turn, is a determining factor in attracting a workforce from abroad [54]. The positive and statistically significant coefficient on the remuneration of physicians indicates that higher wages in the destination country trigger out migration in origin countries. This finding is in line with the argument that better wages represent a major reason for migration in the case of health professionals [59, 60].

We additionally find that the various demand factors are differently correlated with physicians’ brain drain. Greater health spending per capita in destination countries does not have a conclusive effect on the number of migrant arrivals. A distinct picture emerges when we look at health insurance coverage. It seems that a high share of the population covered by health insurance is not necessary favourable for attracting medical doctors from abroad. Our results also reveal that demographic conditions in receiving countries favour increased inflows of medical doctors. This result reinforces the idea that an aging population will increase the demand for health professionals, and will thus intensify migration; since physicians flows are responsive to changes in the share of the elderly population [13]. Similarly, once we control for the supply factors, the number of hospital beds is significantly correlated with immigration flows of medical doctors, indicating that inpatient sector infrastructure is correlated with the demand for medical doctors from abroad.

We also find a positive and highly significant relationship between medical infrastructure and the inflows of foreign-trained doctors. The results reveal that better health care resources in a medical system attracts physicians from abroad.

Taken together, the results of this section suggest that aside from a low unemployment rate and a larger gap in GDP per capita, both the characteristics of the healthcare system, and the population’s needs for healthcare goods in the host country are highly correlated with the utility of migration to the respective country.

### Heterogeneity analysis

Thus far in our analysis, we have focused on the association between migration flows of physicians and various determinants at the destination, regardless of country of origin or time of migration. These overall effects may obscure a differential reaction of physicians to various determinants at the destination between countries of origin. Moreover, given that recent data provides evidence of several disruptions in migration flows, including those of medical doctors, we might expect discrepancies in the relationship between the migration of medical doctors and characteristics of receiving countries. Therefore, the analysis of heterogeneity effects is crucial for acquiring a better understanding of physicians’ brain drain in the last two decades. Table 3 presents the PPML estimates for our preferred specification (full model) for the following key subgroups as sending countries: African countries (column 1), Asian countries (column 2), and Central and Eastern European countries^16^ (column 3). Furthermore, for whole sample, we make a distinction between two time periods: before the economic crisis (2000–2006) and during the economic crisis (2007–2012). It should be noted that the second period selected covers not only the economic crisis and the post-crisis period, but also EU enlargement with the addition of two countries (Romania and Bulgaria), as well as the implementation of the Directive 2005/36/EC on the recognition of professional qualifications, including medical and nursing training.

**Table 3:** Subgroup analysis

Sending regions / time period	Pseudo-Poisson Maximum Likelihood				
	(1)	(2)	(3)	(4)	(5)
	Africa	Asia	Central and Eastern European countries	Before Economic crisis 2000–2006	During Economic crisis 2007–2012
Destination Controls					
Log Unemployment rate [t-1]	−1.627^b^ (0.69)	−2.040^c^ (0.18)	−1.221^c^ (0.27)	−0.258 (0.65)	−2.074^c^ (0.39)
Log PISA Scorereading [t-1]	−3.543 (5.67)	15.252^c^ (4.54)	9.973^c^ (3.62)	7.349 (10.81)	−2.300 (7.15)
Dyadic Controls					
Log GDPo/GDPd [t-1]	1.372 (1.17)	0.200 (0.36)	2.204^c^ (0.69)	0.090 (0.63)	2.652^b^ (1.27)
Log Diaspora [2000]	−0.533^c^ (0.08)	0.573^c^ (0.19)	0.353^c^ (0.06)	0.457^c^ (0.11)	0.461^c^ (0.11)
Log Distance	−0.880^c^ (0.25)	0.026 (0.32)	−0.979^c^ (0.20)	− 0.864^c^ (0.12)	−0.629^c^ (0.12)
Colonial-tie dummy	2.478^c^ (0.29)	1.360^c^ (0.11)	−1.345^c^ (0.21)	0.912^c^ (0.17)	0.463^b^ (0.18)
Common language dummy	1.202^c^ (0.29)	0.826^c^ (0.24)	–	2.082^c^ (0.13)	2.633^c^ (0.16)
Contiguity dummy	0.237 (0.56)	6.143^c^ (0.91)	1.322^c^ (0.23)	0.128 (0.26)	−0.178 (0.17)
Both in EU	–	–	0.145 (0.18)	0.256 (0.21)	0.551^c^ (0.18)
Both in Schengen	–	–	0.028 (0.17)	1.193^c^ (0.23)	0.475^b^ (0.21)
Supply factors					
Log Remuneration of physicians [t-1]	9.001^c^ (3.15)	4.789^c^ (1.39)	−0.558 (1.40)	1.050 (1.88)	6.832^a^ (3.81)
Log Density Physicians per 1000 population [t-1]	−6.829^c^ (1.92)	−3.253^c^ (1.18)	−0.016 (1.11)	0.256 (1.36)	−6.352^b^ (2.73)
Log Medical Graduates per 100,000 population [t-1]	1.078 (0.84)	−0.222 (0.50)	1.580^c^ (0.46)	0.398 (0.41)	2.621^c^ (0.96)
Log Medical Technology [t-1]	4.150^c^ (1.08)	−0.220 (0.55)	1.784^c^ (0.53)	1.012 (0.65)	3.071^c^ (0.78)
Demand factors					
Log public health expenditures [t-1]	−1.844^a^ (1.00)	−0.142 (0.54)	0.720 (0.73)	1.026 (1.30)	−3.593^a^ (2.06)
Log health insurance coverage [t-1]	−6.850^a^ (4.14)	−10.029^c^ (1.79)	2.184 (4.06)	−33.308 (47.68)	9.192 (11.07)
Log Age dependency ratio, old [t-1]	−21.085^c^ (6.81)	−0.458 (3.37)	4.225^c^ (1.40)	8.003^b^ (3.38)	−16.769^c^ (5.74)
Log Hospital beds [t-1]	−4.016^c^ (1.24)	−0.098 (0.38)	2.829^c^ (0.97)	−3.089 (2.03)	1.227 (1.20)
Destination FE	YES	YES	YES	YES	YES
Origin-time FE	YES	YES	YES	YES	YES
Number of clusters (destination*time)	168	153	196	54	78
Observations	4193	4532	2495	6549	9726
R-sqr	0.974	0.978	0.742	0.955	0.787

The table reports PPML estimates of the determinants of international migration in the destination country on the inflow of medical doctors from Africa, Asia and Central and Eastern Europe. The dependent variable represents the number of foreign-trained physicians who have obtained a (partially or fully) registration to practice as medical doctor in the receiving country at time *t*

Estimation period: 2000–2016

Standard errors in parentheses are clustered by destination and time. ^a^, ^b^, ^c^ indicates significance at the 10, 5, and 1% level, respectively

Our heterogeneity analysis reveals that physicians from African countries are particularly attracted to countries that offer higher wages, and by those where the density of medical doctors is relatively low. This result hinges on shortages of medical doctors, which explains the relative attractiveness of that destination for African health professionals. In their case, colonial links play an important role in their choice of destination. Concurrently, higher demand for healthcare services and better medical technology at the destination drive an increase in the inflow of medical doctors from Central and Eastern Europe, whereas Asian medical doctors seem to migrate to countries that have a better school system, offer higher wages, and which have a low density of medical doctors. Destination countries with a higher proportion of elderly individuals and a high number of hospital beds are particularly attractive to medical doctors from Central and Eastern European countries. We also find that the presence of a diaspora favoured migration inflows among physicians only for those coming from Asia or Central and Eastern European countries.

Further analysis shows that better equipped health systems (good remuneration, greater density of medical technology, a younger health industry workforce) were more attractive to foreign medical doctors during the economic crisis than prior to 2007. The results also reveal that between 2007 and 2012, EU member countries experienced 73.50% higher bilateral flows of medical doctors than non-EU countries. We also note that supply factors affecting the healthcare system at the destination played a more important role during the economic crisis than before 2007, with foreign physicians particularly attracted to those countries offering higher wages, better medical infrastructure and a lower density of medical doctors.

All in all, the results in Table 3 demonstrate that country groups and time periods are sources of heterogeneity in the relationship between both migration flows of medical doctors, and pull factors at destinations.

### Robustness

In this section, we provide a set of robustness checks that deal with alternative sample restrictions. Table 4 shows these results based on our preferred specification (column 5, Table 2). First, we restrict our sample by using only 6-year periods (2001, 2004, 2007, 2010, 2013, and 2016). In doing so, we further control for possible endogeneity and mitigate the risk of reverse causality. Secondly, we perform the analysis using data only for the period 2004–2016, and dropping those years for which we have relatively more missing values than for the whole sample.

**Table 4:** Robustness checks

	Pseudo - Poisson Maximum Likelihood	
	(1)	(2)
	2004–2016	6-year period (2001, 2004, 2007, 2010, 2013, 2016)
Destination controls		
Log Unemployment rate [t-1]	−1.278^c^ (0.18)	−0.915^c^ (0.26)
Log PISA Scorereading [t-1]	4.359^a^ (2.43)	4.903 (4.39)
Dyadic Controls		
Log GDPo/GDPd [t-1]	1.796^c^ (0.41)	1.264^c^ (0.47)
Log Diaspora [2000]	0.033^c^ (0.01)	0.032 (0.02)
Log Distance	−0.645^c^ (0.07)	−0.599^c^ (0.12)
Colonial-tie dummy	0.439^c^ (0.10)	0.557^c^ (0.18)
Common language dummy	2.779^c^ (0.11)	2.641^c^ (0.17)
Contiguity dummy	−0.194 (0.13)	−0.188 (0.20)
Both in EU	0.578^c^ (0.13)	0.753^c^ (0.21)
Both in Schengen	0.247^a^ (0.14)	0.319 (0.23)
Supply factors		
Log Remuneration of physicians [t-1]	2.812^c^ (0.83)	1.393 (1.10)
Log Density Physicians per 1000 population [t-1]	−2.420^c^ (0.70)	−2.038^c^ (0.76)
Log Medical Graduates per 100,000 population [t-1]	1.433^c^ (0.43)	0.994^a^ (0.58)
Log Medical Technology [t-1]	0.991^b^ (0.44)	1.057^a^ (0.63)
Demand factors		
Log public health expenditures [t-1]	0.209 (0.51)	0.226 (1.30)
Log health insurance coverage [t-1]	−3.114^a^ (1.84)	−3.835 (2.37)
Log Age dependency ratio [t-1]	2.146^a^ (1.14)	5.279^c^ (1.75)
Log Hospital beds [t-1]	1.304^c^ (0.47)	1.446^a^ (0.85)
Destination FE	YES	YES
Origin-time FE	YES	YES
Number of clusters (destination*time)	172	74
Observations	22,232	9174
R-sqr	0.827	0.846

Standard errors in parentheses are clustered by destination and time. ^a^, ^b^, ^c^ indicates significance at the 10, 5, and 1% level, respectively

The results confirm that our findings are not driven by specific time periods.^17^

### Limitations

There are at least four caveats to the present study. First, time series data provided by the OECD is incomplete, as some countries did not provide relevant information for specific years. However, we succeed in overcoming this shortfall by applying econometric techniques that successfully deal with the presence of a high proportion of zero values in the data. Secondly, the OECD Health Workforce Migration dataset does not capture physicians who may have migrated to other countries, but who are not working as doctors. Thirdly, our dataset does not exclude multiple migrations. It is very likely that medical doctors migrate to more than one destination country in their lifetime. Thus, a country of destination at one point in time might become the country of origin, when a person later decides to make a further move to someplace other than his/her native country. Finally, we have not been able to find detailed information on specific restrictions in destination countries applicable to the medical profession such as language qualifications, additional licensing or certification requirements. Compiling a comprehensive dataset on these requirements and testing their influence on medical migration flows is an area for future research.

## Conclusion

This study contributes to a deeper understanding of the channels through which OECD countries attract medical doctors from abroad. More specifically, this study uses the new module on health worker migration provided by OECD for 2000–2016 in order to examine the determinants of migration flows for foreign-trained physicians. Applying a gravity model approach, and using the Pseudo-Poisson Maximum Likelihood estimator, we find that, apart from dyadic factors, a lower unemployment rate, good remuneration of physicians, an aging population, and a good medical infrastructure in the host country are among the main drivers of physicians’ brain drain. Furthermore, performing an analysis on the mobility of medical doctors from various continents (Africa, Asia and Europe), we find that utility from migration to specific countries may be explained by the heterogeneity of origin countries.

Specifically, we show that physicians from African countries are particularly attracted to destinations offering higher wages, and where the density of medical doctors is relatively low. This result hinges on shortages of medical doctors, which explains the relative attractiveness of that destination to African health professionals. In their case, colonial links also play an important role in the choice of destination. At the same time, higher demand for healthcare services and better medical technology at the destination increase the inflow of medical doctors from Central and Eastern Europe, whereas Asian medical doctors appear to preferentially migrate to countries that have a better school system, and which offer higher wages.

Further analysis shows that better equipped health systems (good remuneration, higher medical technology, young health workforce) were more attractive for foreign medical doctors during the economic crisis than before 2007. We also find that the presence of a diaspora encourages migration inflows among physicians, although only for some specific continents of origin.

Overall, these results draw attention to several factors that affect medical doctors’ decisions to migrate. Policy makers intending to address the personnel shortages in the health care systems could consider these factors when designing strategies to facilitate the entry for foreign physicians, particularly in those countries that have to cope with an aging population and a growing number of people in need of medical services.

## Data Availability

Not applicable.

## Appendix

### List of origin countries

Afghanistan, Albania, Algeria, Angola, Antigua and Barbuda, Argentina, Armenia, Aruba, Australia, Austria, Azerbaijan, Bahamas, Bahrain, Bangladesh, Barbados, Belarus, Belgium, Belize, Benin, Bolivia, Bosnia and Herzegovina, Brazil, Bulgaria, Burkina Faso, Burundi, Cambodia, Cameroon, Canada, Cayman Is- lands, Central African Republic, Chile, China, Colombia, Congo, Costa Rica, Cote d’Ivoire, Croatia, Cuba, Cyprus, Czech Republic, Denmark, Djibouti, Dominica, Dominican Republic, Ecuador, Egypt, El Salvador, Equatorial Guinea, Estonia, Ethiopia, Fiji, Finland, France, Gabon, Georgia, Germany, Ghana, Greece, Grenada, Guatemala, Guinea, Guyana, Haiti, Honduras, Hong Kong, China, Hungary, Iceland, India, Indonesia, Iran, Iraq, Ireland, Israel, Italy, Jamaica, Jordan, Kazakhstan, Kenya, Korea, Kuwait, Kyrgyzstan, Laos, Latvia, Lebanon, Liberia, Libya, Lithuania, Luxembourg, Macedonia, Madagascar, Malawi, Malaysia, Mal- dives, Mali, Malta, Mauritius, Mexico, Mongolia, Morocco, Mozambique, Myanmar, Nepal, Netherlands, New Zealand, Nicaragua, Niger, Nigeria, Norway, Oman, Pakistan, Panama, Papua New Guinea, Paraguay, Peru, Philippines, Poland, Portugal, Qatar, Republic of Moldova, Romania, Russia, Russian Federation, Rwanda, Saint Kitts and Nevis, Saint Lucia, Vincent and the Grenadines, Samoa, Saudi Arabia, Senegal, Seychelles, Sierra Leone, Singapore, Slovak Republic, Slovenia, Somalia, South Africa, Spain, Sri Lanka, Sudan, Suriname, Swaziland, Sweden, Switzerland, Syria, Tajikistan, Tanzania, Thailand, Togo, Trinidad and Tobago, Tunisia, Turkey, Turkmenistan, the United Kingdom, the United States, Uganda, Ukraine, United Arab Emirates, Uruguay, Uzbekistan, Venezuela, Vietnam, Yemen, Zambia, Zimbabwe.

### List of destination countries

Austria, Belgium, Canada, Czech Republic, Denmark, Estonia, Finland, France, Germany, Hungary, Ireland, Israel, Italy, Netherlands, New Zealand, Norway, Slovenia, Spain, Sweden, Switzerland, Turkey, the United Kingdom, the United States.

## Footnotes

1 *Source*: https://stats.oecd.org/Index.aspx?DataSetCode=HEALTH_WFMI

2 *Source*: http://databank.worldbank.org/data/reports.aspx?source=2&series=NY.GDP.PCAP.CD&country=

3 *Source*: https://data.oecd.org/unemp/unemployment-rate.htm

4 *Source*: http://www.oecd.org/pisa/data/

5 Source: http://www.oecd.org/els/mig/dioc.htm

6 Source: http://www.cepii.fr/CEPII/en/bdd_modele/bdd_modele.asp

7 Source: https://data.oecd.org/healthres/doctors.htm

8 Source: https://data.oecd.org/healthres/medical-graduates.htm#indicator-chart

9 Source: https://bit.ly/2qyUrg7

10 Source: https://data.oecd.org/earnwage/average-wages.htm

11 Source: https://stats.oecd.org/Index.aspx?DatasetCode=HEALTH_STAT

12 Source: https://data.oecd.org/healthres/health-spending.htm

13 Source: http://www.oecd.org/els/health-systems/measuring-health-coverage.htm

14 Source: https://data.oecd.org/pop/working-age-population.htm

15 Source: https://data.oecd.org/healtheqt/hospital-beds.htm

16 Bulgaria, Czech Republic, Estonia, Greece, Hungary, Latvia, Lithuania, Poland, Republic of Moldova, Romania, Slovak Republic, Slovenia, and Ukraine.

17 We additionally perform a LINK test [61] in order to check evidence for miss-specification of our gravity full model (results are available upon request). This test provides no evidence for miss-specification of our gravity full model.
